# Phelan-McDermid Syndrome in Spanish children: gastrointestinal manifestations in relation to nutritional intake

**DOI:** 10.3389/fnut.2025.1668101

**Published:** 2025-10-16

**Authors:** Sandra Carrera-Juliá, María Jesús Vega-Bello, Bárbara Gómez-Taylor, Eraci Drehmer, Mari Ángeles Navarro, Mari Luz Moreno

**Affiliations:** ^1^Department of Nutrition and Dietetics, Universidad Católica de Valencia “San Vicente Mártir”, Valencia, Spain; ^2^Department of Human Anatomy and Physiology, Universidad Católica de Valencia “San Vicente Mártir”, Valencia, Spain; ^3^Department of Health Sciences, Universidad Católica de Valencia “San Vicente Mártir”, Torrente, Spain; ^4^Department of Basic Sciences, Universidad Católica de Valencia “San Vicente Mártir”, Torrente, Spain

**Keywords:** Phelan-McDermid Syndrome, macronutrients, micronutrients, nutritional intake, growth, genetic disease, rare disease

## Abstract

**Background:**

Phelan-McDermid Syndrome (PMS) is a rare genetic disorder associated with neurodevelopmental delay, speech impairment, and frequent gastrointestinal symptoms. While dietary management may influence health outcomes in rare diseases, there is limited data on the nutritional profile of PMS patients. This study aimed to evaluate dietary intake, digestive symptoms, and growth parameters in Spanish children with PMS.

**Methods:**

A cross-sectional descriptive study was conducted involving 37 Spanish children (aged 1–18 years) diagnosed with PMS. Digestive symptoms and food intolerances were collected through patient histories. Dietary intake was assessed using a 7-day food diary and a Food Frequency Questionnaire, analyzed with Easy Diet^®^ software. Nutritional adequacy was evaluated against national (SENC, FESNAD) and international (WHO) recommendations. Anthropometric measurements (weight, height and BMI) were taken according to ISAK standards.

**Results:**

Most participants (75.7%) presented digestive symptoms, with significant gender differences observed in swallowing difficulties, reflux, and gases. Diet analysis revealed significant deficiencies in energy, fiber, calcium, iron, vitamins D and E and excesses in simple sugars, protein, saturated fats, cholesterol, and certain vitamins and minerals. Anthropometric data showed mean weight, height, and BMI around the 50th percentile and mean WHO z-scores were close to the reference median (WAZ = 0.03, HAZ = −0.15, BAZ = 0.16), supporting an overall adequate growth pattern.

**Conclusion:**

Given the gastrointestinal symptoms observed in Spanish children with PMS, specialized nutritional supervision is required, whereby nutrition professionals educate children, parents, and caregivers on strategies such as increasing fibre and calcium/vitamin D intake, with supplementation when required, and limiting simple sugars, saturated fats, and processed meats. These interventions aim to address the identified imbalances and enhance the quality of life of individuals with PMS.

## 1 Introduction

According to OMIM, Phelan-McDermid Syndrome (PMS) can be caused by a heterozygous contiguous gene deletion at chromosome 22q13 or by mutation in the SHANK3 gene (606230), which is located within the minimum critical region (1). The size of the deleted segment ranges from less than 100 kb to more than 9 Mb (2, 3). Within this region, there are over 90 genes, but the critical region identified for the manifestation of the syndrome is an area of approximately 100 kb that primarily contains three genes: *ACR, SHANK3* and *RABL2B*, of which *SHANK3* is considered the hallmark gene for the development of Phelan-McDermid Syndrome (4, 5). The deficiency in *SHANK3* gene activity leads to a reduced number of dendrites and an imbalance in synaptic transmission (6). This deficit is associated with severe developmental alterations that affect multiple organ systems to different severity degrees (7–9).

PMS is underdiagnosed, and its true incidence remains unknown. By 2020, approximately 2,000 cases had been reported worldwide, around 201 of which were in Spain. In recent years, its incidence in Spain has been increasing, with 25 to 35 new cases reported annually (10). The syndrome occurs equally in women and men and can be inherited or *de novo* (80% of cases) (9, 11).

The most characteristic clinical symptoms are global developmental delay, absent or severely delayed speech and morphological alterations such as long eyelashes, large or prominent ears, bulbous nose, pointed chin, fleshy hands, and dysplastic toenails (1). Other symptoms that may be present are related to structural brain abnormalities, seizures, motor deficits, hypotonia, and, to varying degrees of severity, autism spectrum disorder (1–4). Gastrointestinal symptoms are common in PMS and include gastroesophageal reflux disease, constipation, and diarrhea (12–15). Cyclical vomiting has also been reported in some patients (15, 16). Eating difficulties are also common (2, 13) and may be related to low muscle tone.

Despite the benefits that an appropriate diet can provide in supporting favorable outcomes in rare diseases (17, 18), evidence on nutritional intake in such populations is scarce. Previous research has shown that children with intellectual disability and autism spectrum disorders often present suboptimal dietary patterns, including high intake of saturated fat and sugars and insufficient consumption of fiber, vitamins, and minerals, which may exacerbate gastrointestinal symptoms, metabolic risk, and behavioral difficulties (19, 20). Considering that Phelan-McDermid Syndrome shares clinical features with autism spectrum disorders, it is plausible that similar nutritional challenges may be present in this group. Therefore, the present study analyzes the nutritional intake of a group of Spanish patients under 18 years of age diagnosed with PMS. Additionally, clinical digestive symptoms and anthropometric measurements are evaluated with the aim of correlating them with the aforementioned factors. While exploratory in nature, this study was guided by the expectation that children with PMS might present imbalances consistent with or greater than those reported in other neurodevelopmental disorders, underscoring the importance of targeted nutritional assessment in this population.

## 2 Materials and methods

### 2.1 Type of study

A descriptive cross-sectional study in humans was performed in order to study the nutritional intake of a group of Spanish children diagnosed with PMS and its influence on the condition of their digestive system and growth.

### 2.2 Characteristics of participants

This study was conducted with a population-based sample of 37 Spanish children aged 1–18 years, of whom 19 were girls and 18 were boys, all diagnosed with PMS. The 37 patients were recruited exclusively through the Phelan-McDermid Syndrome Association of Spain, which served as a bridge to the affected families. Participants provided medical reports of the tests they underwent at hospitals or medical centers. The inclusion criteria were: age below 18 and diagnosed with PMS that were able to eat orally without the need for enteral or parenteral nutrition. Exclusion criteria included patients using feeding tubes, those with other rare or concomitant neurological diseases, and those whose caregivers were unable to complete the dietary questionnaires. The study was conducted according to the guidelines of the Declaration of Helsinki (21) and approved by the Human Research Ethics Committee of the Universidad Católica de Valencia “San Vicente Mártir” (procedure number UCV/01819-108). All participants and their parents signed a written informed consent form containing details on the procedures and nature of the study.

### 2.3 Digestive status

In addition to the sociodemographic information of the patient (age, sex, etc.), the presence of food intolerances and digestive symptoms were registered from the patient’s clinical history. The term “digestive symptoms” is used as a general term to represent any type of manifestation that causes discomfort or distress in the patient’s gastrointestinal tract. On the other hand, specific digestive symptoms (bad breath, swallowing difficulties, gases and bloating, reflux, constipation and diarrhea) were measured.

### 2.4 Assessment of dietary intakes

The Food Frequency Questionnaire was used (22) to gather information about how often the different food groups were consumed by the children: dairy products, fruits, vegetables, meat, fish, rice, legumes, pasta, eggs, juices, nuts, seafood, snacks, sweets, etc. The self-administered questionnaire asked about how often certain food groups were usually consumed in a week. In addition, parents of each child registered their solid and liquid food intake for 7 days by a dietary diary. This period allowed us to collect enough data of the children’s normal diet, reducing the risk of bias associated with choosing 1 day a week (23). Parents also took notes of the type of food they consumed, the different ingredients used to prepare each dish and the amount that was consumed per intake (a glass, a portion, a cup, a plate, etc.) or the exact weight of the food or drink. The use of dietary supplements was excluded from the nutrient intake analysis because our aim was to determine the quality and pattern of the patients’ diet without taking into account additional contributions such as the use of supplements. In order to help parents to complete the task, they were provided with information regarding the weight for each portion and the most common household measurements (24). Once a week, the patients’ caregivers were phoned and asked to report on how they were completing the FFQs and food diaries. During these phone calls, the caregivers explained how they were recording the data and our nutritionist-dieticians from the research group answered any questions they raised. In addition, caregivers were instructed to record meals on a daily routine so that nothing would be forgotten. In some cases, caregivers even sent photos of meals to verify whether the amount on the plate matched the grams they had recorded in the dietary diary.

With the information of the children’s food diary over 7 days and the Food Frequency Questionnaire, the quality of the diet was calibrated using the Easy Diet-Programa de gestión de la consulta^®^ software (25). With this software, a nutrition profile containing the daily energy intake and percentage distribution of macronutrients (proteins, lipids and carbohydrates) and micronutrients (vitamins and minerals) was obtained from the meals introduced into the program.

Additionally, to assess whether the diet was adequate or not, the nutritional guidelines from the Sociedad Española de Nutrición Comunitaria (SENC) and Federación Española de Sociedades de Nutrición, Alimentación y Dietética (FESNAD) at the national level (26, 27), as well as the recommendations established by the World Health Organization (WHO), were used as references. The nutritional adequacy index (AI) was calculated as the ratio between the observed intake of a nutrient and the recommended intake, expressing the result as a percentage. AI is an indicator that measures whether the intake of a specific nutrient meets the dietary recommendations established for a given population. Values above 100% would indicate an excess intake of that nutrient, while values below 100% would indicate a deficiency.

### 2.5 Assessment of anthropometric parameters

Anthropometric assessments in the study adhered to the International Society for the Advancement of Kinanthropometry (ISAK) protocols (28). All measurements were conducted using validated and calibrated equipment. Measurements were taken by a certified ISAK level III anthropometrist.

A SECA (Hamburg, Germany) portable clinical scale was used to measure body weight. This scale has a range of 150–200 kg and a precision of 100 g, ensuring accurate weight measurements. To accurately measure height to a precision of 0.1 cm, a SECA 220 model stadiometer was employed. Body Mass Index (BMI) was calculated as weight (kg) divided by height squared (m^2^). Reference percentiles for Spanish children were obtained from the Spanish Society of Endocrinology and Nutrition (SEEN) according to sex and age (29).

In addition, weight-for-age (WAZ), height-for-age (HAZ) and BMI-for-age (BAZ) z-scores were calculated according to the World Health Organization (WHO) growth standards. For children younger than 5 years, it was used the WHO 2006 Child Growth Standards, and for participants aged 5–19 years, the WHO 2007 Growth Reference (AnthroPlus). Cutoffs were: WAZ: underweight = z < −2 SD; severe underweight = z < −3 SD; HAZ: stunting = z < −2 SD; severe stunting = z < −3 SD; BAZ: thinness = z < −2 SD; severe thinness = z < −3 SD; overweight = z > +1 SD; obesity = z > +2 SD.

### 2.6 Statistical analysis

The results are presented as mean ± standard deviation, number of patients or percentage relative to the total sample number. Data were analyzed using Microsoft Excel and Statgraphics Centurion XVII software (Stat Point, Inc., Herndon, Virginia 187, United States). Statistical analysis was performed with SPSS software v.23 (IBM Corporation, Armonk, NY, United States). When the data followed a normal distribution, Student’s *t* tests were applied for comparisons between two groups; otherwise, the Mann–Whitney U test was used. For categorical variables, chi-square tests were performed. Due to the small sample size in some categories, Fisher’s exact test was applied instead of the chi-square test. No missing data were identified in the main variables, as the food diaries were reviewed with the families to ensure completeness prior to analysis. A *p*-value below 0.05 was considered statistically significant. Given the exploratory nature of the study, no corrections for multiple comparisons were applied, and results should be interpreted with caution.

The results are presented as mean ± standard deviation, number of patients, or percentage relative to the total sample size. Data were analyzed using Microsoft Excel and Statgraphics Centurion XVII software (Stat Point, Inc., Herndon, VA, United States). Statistical analyses were performed with SPSS software v.23 (IBM Corporation, Armonk, NY, United States). The distribution of variables was assessed using the Kolmogorov–Smirnov test. When the data followed a normal distribution, Student’s *t* tests were applied for comparisons between two groups; otherwise, the Mann–Whitney U test was used. For categorical variables, chi-square tests were performed. No missing data were identified in the main variables, as the food diaries were reviewed with the families to ensure completeness prior to analysis. A *p*-value below 0.05 was considered statistically significant. Given the exploratory nature of the study, no corrections for multiple comparisons were applied, and results should be interpreted with caution.

## 3 Results

### 3.1 General characteristics of the patients

The study sample consisted of a total of 37 patients, of whom 18 were boys and 19 were girls, representing 48.6 and 51.4%, respectively. The mean age of the sample was 9.5 ± 5.6 years.

The study sample was stratified by developmental stage (Table 1). The preschool stage (1–5 years) included 9 patients, representing 24.3% of the total sample. The school-age group (6–9 years) comprised 11 patients, accounting for 29.7% of the total. The pre-adolescent and adolescent stage (10–19 years) consisted of 17 patients, representing 45.9% of the sample.

**Table 1:** Distribution of the study sample by sex and age.

Stage	Total		Girls		Boys	
	*N*	%	*N*	%	*N*	%
Preschool (age 1–5)	9	24.3	4	21.1	5	27.8
School (age 6–9)	11	29.7	6	31.6	5	27.8
Pre-adolescent and adolescent (age 10–19)	17	45.9	9	47.3	8	44.4

No statistically significant differences were identified with respect to sex, suggesting a comparable distribution of girls and boys with PMS in our sample.

### 3.2 Digestive symptoms

The analysis of digestive symptoms (Table 2) revealed that the number of patients presenting such symptoms was significantly higher (*p* = 0.001) than those who did not report any digestive-related issues (Table 2). Specifically, a total of 28 patients (75.7%) reported discomfort affecting the digestive system, compared to 9 patients (24.4%) who did not. Among those who experienced symptoms, there were 14 girls (73.7%) and 14 boys (77.8%).

**Table 2:** Different digestive symptoms presented by the patients in the study sample.

		Total		Girls		Boys	
		*N*	%	*N*	%	*N*	%
Digestive symptoms** (*p* = 0.001)	Yes	28**	**75.7**	14	73.7	14	77.8
	No	**9**	**24.3**	5	26.3	4	22.2
Food intolerances	Yes	10	27	5	26.3	5	27.8
	No	27	73	14	73.7	13	72.2
Bad breath	Yes	14	37.8	7	36.8	7	38.9
	No	23	62.2	12	63.2	11	61.1
Swallowing difficulties	Yes	4	10.8	**1**	**5.3**	**3**^**#**^ **(*p* = 0.042)**	**16.7**
	No	33	89.2	**18**	**94.7**	**15**	**83.3**
Reflux	Yes	10	27	**8**^**#**^ **(*p* = 0.020)**	**42.1**	**2**	**11.1**
	No	27	73	**11**	**57.9**	**16**	**88.9**
Gases and bloating	Yes	8	21.6	**6**^**#**^ **(p = 0.048)**	**31.6**	**2**	**11.1**
	No	29	78.4	**13**	**68.4**	**16**	**88.9**
Constipation	Yes	12	32.4	7	36.8	5	27.8
	No	25	67.6	12	63.2	13	72.2
Diarrhea	Yes	5	13.5	3	15.8	2	11.1
	No	32	86.5	16	84.2	16	88.9

The term digestive symptoms is used as a general term to represent any type of manifestation that causes discomfort or distress in the patient’s gastrointestinal tract. On the other hand, specific digestive symptoms (bad breath, swallowing difficulties, gases and bloating, reflux, constipation and diarrhea) were measured. Fisher’s exact test was applied in this statistical analysis. That is why the number of patients with single symptoms do not add up to the total 28 The statistical difference is indicated as ** *p* ≤ 0.01; * *p* ≤ 0.05 when the presence (yes) of a symptom was significantly higher than the absence (no). In addition, statistical differences ^##^ *p* ≤ 0.01; ^#^ *p* ≤ 0.05 between boys and girls are shown. Significant data are highlighted in bold.

Regarding food intolerances, 10 patients (27%) reported, whereas 27 patients (73%) did not. Lactose intolerance was identified as the most frequent among the reported cases.

As for the presence of bad breath, 14 patients reported this symptom representing 37.8%. When analyzing swallowing difficulties, 4 patients (10.8%) reported experiencing such problems. A total of 10 patients (27%) were found to suffer from gastroesophageal reflux. Regarding gases and bloating, these symptoms were reported by 8 patients (21.6%). A total of 12 patients (32.4%) suffered from constipation. Finally, the analysis of diarrhea indicated that 5 patients (13.5%) experienced it. Although the tests performed did not reach statistical significance, percentages of 27–37.8%, which represent around one third of the sample, could arguably be clinically significant, even if not statistically significant. It should be added that food intolerances, bad breath, swallowing difficulties, reflux, gases and bloating, constipation, and diarrhea could be influenced and aggravated by the poor eating habits observed (see section 3.3).

Regarding gender-based differences, Fisher’s exact test was applied, and statistically significant differences were observed in the variables swallowing difficulties (*p* = 0.042), reflux (*p* = 0.020) and gases (*p* = 0.048). Specifically, boys experienced greater swallowing difficulties compared to girls, whereas the opposite was observed in the case of gastroesophageal reflux and the presence of gases.

### 3.3 Nutritional intakes

#### 3.3.1 Macronutrients

A study was conducted on the habitual dietary intake of the patients (Table 3), analyzing the following variables: energy, macronutrients (carbohydrates, proteins, and lipids), lipid profile, cholesterol, and fiber.

**Table 3:** Description of the average daily nutritional intake of macronutrients in the study sample.

Nutritional variable	AI (%)		*p* (1) RDA WHO	*p* (2) RDA SENC
	Mean	SD		
Energy (Kcal/day)	76.75	14.98	0.000**	–
Carbohydrates (%/day)	74.63	9.22	0.000**	–
Simple carbohydrates (%/day)	486.15	123.04	0.000**	–
Proteins (%/day)	240.28	77.03	0.000**	–
Lipids (%/day)	114.16	10.98	–	0.000**
MUFAs (%/day)	209.15	20.67	–	0.000**
PUFAs (%/day)	263.15	47.58	–	0.000**
SFAs (%/day)	416.22	70.86	–	0.000**
Cholesterol (mg/day)	149.96	49.16	–	0.000**
Fiber (g/day)	15.36	5.03	–	0.000**

AI, Adequacy Index; RDA, Recommended Dietary Allowance; MUFA, Monounsaturated Fatty Acids; PUFA, Polyunsaturated Fatty Acids; SFA, Saturated Fatty Acids; WHO, World Health Organization. SENC: Spanish Society of Community Nutrition. p (1) compared with WHO RDA recommendations. p (2) compared with SENC recommendations. The Dietary Reference Intake (DRI) for each nutrient analyzed is not presented in the table, as the values differ according to the sex and age range of each patient in the study sample. *t*-Student test was applied in this statistical analysis. The statistical difference is indicated as ** *p* ≤ 0.01.

Based on the obtained data and after analyzing the daily diet and eating habits of the study population, the mean adequacy index (AI) for daily energy was 76.75%. In terms of carbohydrates intake, it was 74.73% of the recommendations while the mean adequacy index of simple carbohydrates was 486.15%. Regarding proteins, the AI was 240.28%. In the group of lipids, the AI of fatty acids was 114.16%. More specifically, the AI of monounsaturated fatty acids was 209.15%, from polyunsaturated fatty acids was 263.15% and from saturated fatty acids was 416.22% compared to recommendations. Finally, the mean AI for cholesterol was 149.96 and 15.36% for fiber (Table 3).

The adequacy indices separated by gender (boys and girls) are not shown in Table 3, as no significant differences were found between sexes.

In summary, the amount of energy provided by the diet, along with carbohydrates and fiber, was significantly lower (*p* = 0.000) than the recommended levels, with fiber being particularly notable, as its intake was less than one-fifth of the recommended amount. In contrast, the intake of simple sugars, proteins, MUFAs, PUFAs, SFAs, and cholesterol exceeded significantly (*p* = 0.000) dietary recommendations in all cases, with some reaching levels up to four times higher.

#### 3.3.2 Micronutrients

Table 4 presents the summarized data the average daily nutritional intake of micronutrients in the study sample. The mean AI of vitamin B1 was 145.12% of the WHO recommendations. The AIs for niacin and folic acid were 147.48 and 75.03%, respectively. Similarly, the adequacy index for vitamin B12 was 243.28%, and, in the same line, vitamin C showed an AI of 240.04%. In contrast, the intakes of vitamins D and E were below the recommended levels, with adequacy indices of 44.74 and 84.90%, respectively. The AIs of these vitamins and minerals according to FESNAD recommendations are presented in Table 4 and follow a similar pattern to those based on WHO guidelines.

**Table 4:** Description of the average daily nutritional intake of micronutrients in the study sample.

Nutritional variable	AI % (WHO)			AI % (FESNAD)		
	Mean	SD	p (1) RDA WHO	Mean	SD	p (2) RDA FESNAD
Vit B1-Thiamine (mg/day)	145.12	46.87	0.000**	144.13	44.34	0.000**
Vit B3-Niacin (mg/day)	147.48	44.68	0.000**	141.71	30.05	0.000**
Vit B9-Folic acid (μg/day)	75.03	37.20	0.000**	112.26	59.40	0.218
Vit B12-Cyanocobalamin (μg/day)	243.28	94.79	0.000**	320.6	121.94	0.000**
Vit C-Ascorbic acid (mg/day)	240.04	125.00	0.000**	176.71	87.81	0.000**
Vit D (μg/day)	44.74	27.16	0.000**	42.96	27.81	0.000**
Vit E (mg/day)	84.90	26.01	0.001**	67.89	24.58	0.000**
Calcium (mg/day)	87.70	33.66	0.033*	86.50	24.77	0.002**
Iron (mg/day)	85.20	24.74	0.001**	93.33	29.15	0.173
Magnesium (mg/day)	204.21	107.00	0.000**	139.42	66.26	0.001**
Phosphorus (mg/day)	158.18	57.69	0.000**	174.97	38.93	0.000**
Zinc (mg/day)	–	–	–	110.46	27.81	0.028*

AI, Adequacy Index; RDA, Recommended Dietary Allowance; FESNAD, Spanish Federation of Nutrition Societies; Vit, Vitamin. p (1) compared with WHO RDA recommendations. p (2) compared with FESNAD recommendations. WHO: World Health Organization. The Dietary Reference Intake (DRI) for each nutrient analyzed is not presented in the table, as the value varies according to the sex and age range of each patient in the study sample. *t*-Student test was applied in this statistical analysis The statistical difference is indicated as ** *p* ≤ 0.01; * *p* ≤ 0.05.

Regarding minerals, calcium and iron intakes were insufficient, corresponding to 87.70 and 85.20% of the WHO recommendations, respectively. In contrast, magnesium and phosphorus intakes were excessive, reaching 204.21 and 158.18% of the recommended levels. For zinc, an adequacy index of 110.46% was observed according to FESNAD recommendations.

Similarly, to what was observed with macronutrients, the adequacy indices separated by gender (boys and girls) are not shown, as no significant differences were found between sexes.

In summary, intakes of vitamins B1, B3, magnesium, phosphorus, and zinc were significantly higher than the recommended levels (*p* = 0.000, except for zinc, *p* = 0.028). Vitamins B12 and C were particularly notable, with intakes more than double the recommended amounts. In contrast, intakes of vitamin E, calcium, and iron were significantly lower than dietary recommendations (*p* = 0.001, *p* = 0.033, and *p* = 0.001, respectively). Vitamin D deficiency was especially pronounced, with intake levels falling below half of the recommended amount.

### 3.4 Growth

Weight, height, and BMI, as well as their corresponding percentiles, were assessed in the studied population in order to determine whether the children were growing normally.

**Figure 1:**
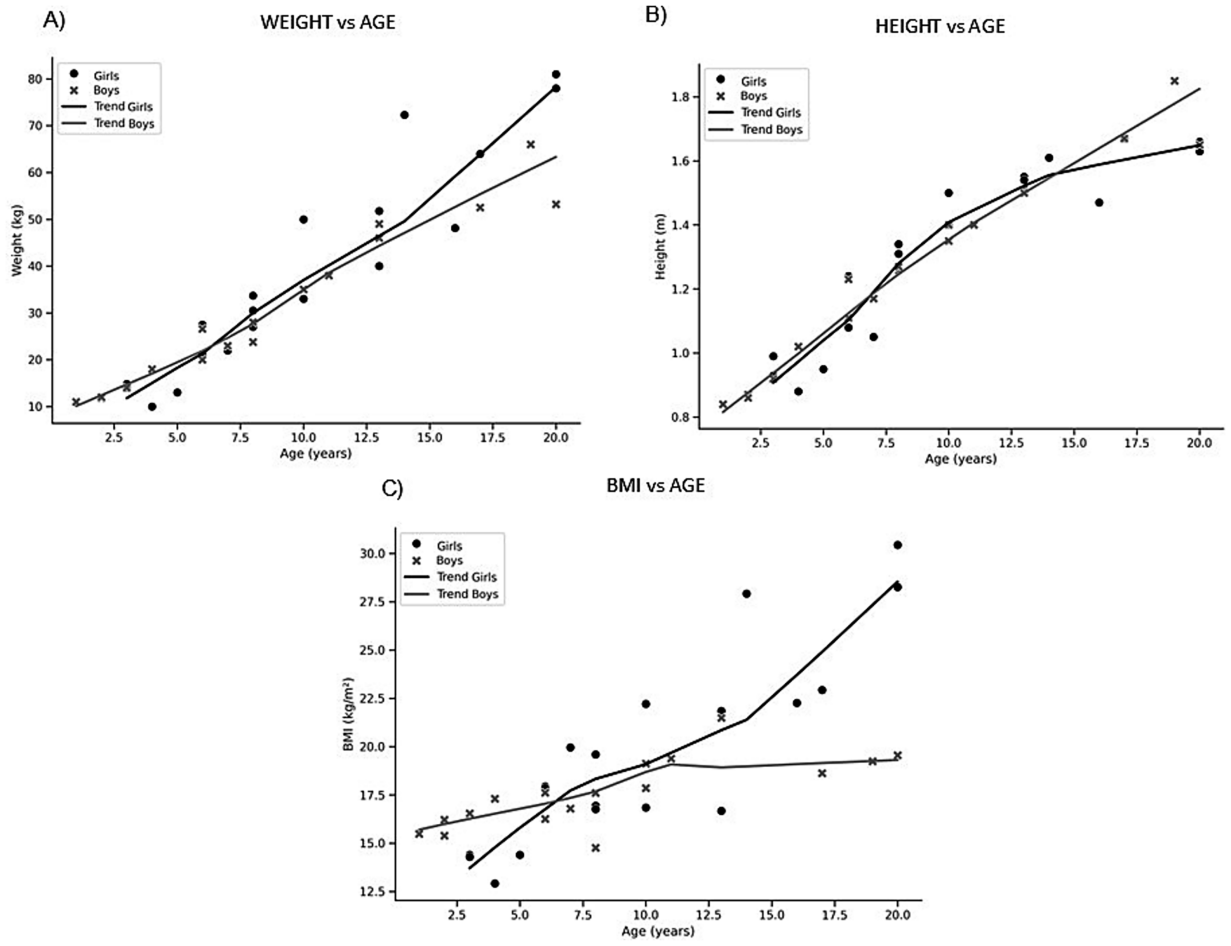
Scatterplots of anthropometric variables according to age and sex. Individual values plotted against age in girls (black circles) and boys (grey crosses). **(A)** Weight vs. age; **(B)** Height vs. age. **(C)** BMI vs. age.

The mean weight of the children was 34.99 ± 19.92 kg, with no significant differences observed between the mean weight of boys and girls. Regarding weight percentiles, the group was around the 50th percentile (49.84 ± 20.96%), indicating an adequate weight. However, significant differences were observed between boys and girls (*p* = 0.0311), with girls exceeding the 50th percentile (approximately 55%), while boys remained below it (approximately 45%). Figure 1A shows individual weight values plotted against age. In girls, weight ranged from 10 to 81 kg (mean 38.5 ± 22.5 kg), while in boys the range was 11 to 66 kg (mean 31.3 ± 16.6 kg). Both sexes exhibited a progressive increase in body weight with advancing age. The dispersion of values widened during adolescence, reflecting greater interindividual variability. Although the trajectories were similar, girls tended to present higher weight values at older ages.

In terms of height, the mean stature was 1.30 ± 0.82 m, with no significant differences between sexes. Regarding height percentiles, the group presented values close to the 50th percentile (46.46 ± 21.87%), suggesting normal height. In this case, no significant differences between boys and girls were found. Figure 1B presents the distribution of height by age. Girls showed heights between 0.88 and 1.67 m (mean 1.32 ± 0.22 m), whereas boys ranged from 0.84 to 1.85 m (mean 1.27 ± 0.27 m). A steady increase in stature was observed in both sexes throughout childhood and adolescence. After approximately 10 years of age, the dispersion became more pronounced, consistent with the onset of pubertal growth. Overall, boys tended to display slightly greater average height values compared with girls during later adolescence.

Finally, the BMI of the children was 18.72 ± 3.95 kg/m^2^, indicating a generally normal weight status for the population. However, when analyzed by sex, significant differences were found (*p* = 0.0001): boys had a slightly underweight average BMI (17.61 ± 1.79 kg/m^2^), whereas girls had a higher mean BMI. BMI percentiles did not show significant differences between sexes, with the total population averaging around the 50th percentile (54.49 ± 22.21%). Figure 1C depicts BMI according to age. In girls, BMI ranged from 14.4 to 30.5 kg/m^2^ (mean 19.7 ± 5.0), while in boys values extended from 14.8 to 21.5 kg/m^2^ (mean 17.6 ± 1.8). BMI values remained relatively stable during childhood, with a moderate upward trend during adolescence. Girls displayed greater variability, with several individuals exceeding 25 kg/m^2^, while boys presented a narrower distribution. These findings suggest comparable BMI trajectories across sexes, but with higher dispersion among girls.

Based on WHO growth standards, none of the participants were classified as underweight (WAZ < −2 SD) or stunted (HAZ < −2 SD). Likewise, no cases of severe thinness (BAZ < −3 SD) or thinness (BAZ < −2 SD) were observed. However, six children were classified as overweight (BAZ > +1 SD), while no participants met the criteria for obesity (BAZ > +2 SD). When stratified by sex, mean z-scores were similar between boys and girls. Boys had a mean WAZ of −0.15, compared to +0.19 in girls (*p* = 0.13). Mean HAZ was −0.07 in boys and −0.22 in girls (*p* = 0.54). For BAZ, boys averaged −0.05 and girls +0.37, with a trend toward higher values in girls (*p* = 0.078), although this difference did not reach statistical significance.

In summary, Spanish children with Phelan-McDermid Syndrome appear to experience normal growth despite their condition and suboptimal dietary habits.

## 4 Discussion

To date, no studies in Spain have thoroughly examined the nutritional intake of patients with PMS. This study is therefore important as it is the first to jointly describe nutritional intake patterns, digestive symptoms, and growth in Spanish children affected by this disorder.

In the analyzed sample, no significant sex differences were found in prevalence, suggesting an equal distribution between boys and girls in our sample. This finding is consistent with previous studies, which estimated a prevalence of 0.4/10,000 inhabitants (30), with no significant differences by sex (10).

### 4.1 Digestive symptomatology and nutritional intake

Previous evidence has indicated that digestive problems are common in patients with PMS and can negatively impact children’s quality of life (31). Our results are in line with this, as a significantly higher number of patients exhibited digestive symptoms compared to those who did not. Notably, lactose intolerance was the most frequently reported symptom, which may contribute to the intestinal discomfort observed.

It was found that calcium intake was significantly lower than the established recommendations, a finding potentially related to the prevalence of lactose intolerance in our sample. Since dairy products (such as milk, yogurt and cheese) are a primary dietary source of calcium, children following a lactose-restricted diet may be predisposed to inadequate calcium intake (32). Individual interviews with parents and caregivers revealed that, in most cases, lactose restriction was managed by avoiding dairy products rather than consuming lactose-free alternatives. Consequently, not only were traditional dairy sources of calcium eliminated, but children also did not compensate with other calcium-rich, lactose-free foods due to lack of awareness. This suggests a potential relationship between lactose intolerance and lower calcium intake in this population.

Although linear growth was within normal ranges, inadequate calcium intake may affect bone mineral density more than stature (33). This hypothesis should be confirmed through specific bone density studies in the population analyzed.

More than half of patients with PMS present chewing and swallowing difficulties, secondary to muscle hypotonia and dental abnormalities such as malocclusion, diastemas, and high-arched palate (34). However, in this study, only four children were identified with swallowing difficulties. This may be because all participants could feed orally, without needing major dietary modifications for texture or other adaptations commonly used in patients with dysphagia. No significant differences were found between participants with and without gastroesophageal reflux, a commonly reported symptom in individuals with PMS (35). Similar results were observed for gas and bloating, even though these symptoms are considered characteristic of the syndrome.

In addition to the reduced sample size, a possible explanation for the lack of statistically significant associations may relate to differences in nutritional management among families. Although our study did not collect data on caregiver education or professional guidance, it is possible that some families may implement dietary strategies to reduce digestive symptoms, such as avoiding gas-producing foods or foods associated with reflux. This variability could influence both the prevalence of digestive symptoms and nutritional intake, but further research is needed to confirm these potential associations. Clarifying these factors in future studies could also help understand observed differences in symptom patterns between sexes.

With regard to constipation, various factors associated with PMS (such as gastrointestinal hypomotility, impaired defecation control, inadequate hydration, the use of certain medications, and reduced mobility) may contribute to its occurrence (35). However, no statistically significant differences in constipation were observed in our study. Nonetheless, a deficient fiber intake was identified in the sample, which may be a relevant underlying factor, given the evidence that adequate dietary fiber intake can reduce constipation in the pediatric population (36). A similar interpretation can be made for cases of diarrhea. In this line, the clinical repercussions of constipation and, particularly, diarrhea may include abdominal pain, digestive discomfort, incontinence, risk of dehydration, and nutrient malabsorption. Therefore, the presence of constipation and/or diarrhea has clear clinical implications, which may even contribute to a loss of autonomy when patients experience them severely.

### 4.2 Growth and nutritional intake

The results of our study show that Spanish children with PMS exhibit average anthropometric parameters close to the 50th percentile of reference values. However, when analyzing percentils in detail, it was observed that girls slightly exceeded the 50th percentile in weight and BMI, while boys fell below it. These differences between boys and girls have also been observed in children with autism spectrum disorder (ASD) (37, 38). Taken together, these findings suggest overall adequate growth in this cohort of children with PMS.

This pattern of normal growth is consistent with the classical clinical description of PMS. The literature emphasizes that normal stature is a common feature in PMS (39). Previous studies indicate that the majority of these patients fall within normal growth ranges. For example, it is reported that approximately 78% of individuals with PMS had height within the normal range (with only 11% below the 5th percentile and 11% above the 95th) (39). Similarly, Schön et al. reported normal postnatal growth in most cases, with only 10% of patients below the 3rd percentile for height and 20% above the 95th, based on their literature review (40). In our study, weight and height percentiles were located near the 50th percentile, supporting the conclusion that children with PMS grow normally. Only a minority showed extreme values, highlighting the absence of a generalized growth delay pattern.

Several explanations may account for this apparent paradox (growth parameters are normal even with the suboptimal diets). First, it is possible that compensatory physiological mechanisms help maintain adequate growth, even in the presence of suboptimal nutrient intake, at least in the short term. Second, parental adaptations in feeding practices may play a role: families often modify diets to ensure adequate caloric intake or to avoid foods that exacerbate gastrointestinal symptoms, which may inadvertently balance some deficits. Third, we cannot exclude that some nutrient deficiencies were overestimated due to limitations inherent to dietary assessment tools, particularly given the reliance on caregiver-reported food diaries. While growth appears normal in the studied cohort, nutritional imbalances may still exert negative health effects over time, underscoring the need for longitudinal follow-up.

The literature shows that most children with PMS are not overweight, and as adults they usually remain within a normal weight range. However, about 10% gain excessive weight due to inactivity or compulsive eating (39). Our findings regarding average BMI also fall within the expected range for age, indicating a generally normal growth.

An interesting finding in our study is the sex-based difference: girls showed slightly higher weight percentiles and a significantly higher BMI compared to boys (*p* = 0.0001). The literature lacks clear reports on sex differences in growth among individuals with PMS, making this result novel. One possible explanation is pubertal maturation. Precocious puberty has been documented in approximately 13% of patients with PMS and is more common among girls (14, 41). This early onset of puberty could result in a relative increase in weight and body fat at an early age. Nonetheless, these differences could also be due to sample variability.

These findings highlight that Spanish children with PMS in our cohort show overall normal growth, despite digestive and feeding issues. Girls tended to show slightly higher body measurements compared to boys, possibly due to hormonal or maturational effects (40, 41), which warrants further investigation. In clinical practice, these findings underscore the importance of carefully monitoring the nutritional status of patients with PMS (weight, height, BMI, dietary intake) at each visit. This approach ensures optimal growth and early detection of any deviations, helping guarantee that (even with restrictive diets) children reach their developmental potential.

### 4.3 Other consequences of poor nutritional intake

The analysis of the patients’ nutritional intake and its comparison with the DRIs revealed that the diet of the children was calorically inadequate and unbalanced in terms of macronutrient and micronutrient distribution, which could compromise the nutritional density of the diet.

In particular, dietary fiber intake was extremely low (approximately 15% of the recommended amount), likely related to the insufficient consumption of plant-based foods (fruits, vegetables, legumes, nuts, among others), as observed in the analysis of the 7-day dietary records and the food frequency questionnaire. This finding is noteworthy because fiber consumption may be linked to digestive symptoms (such as constipation) and the configuration of the gut microbiota. Scientific evidence suggests that intestinal microbiota is altered in patients with PMS. Specifically, a Spanish study comparing the fecal microbiota of children with PMS and healthy controls found significant differences in bacterial composition and levels of short-chain fatty acids (SCFAs) (42). In particular, a lower abundance of key bacterial genera such as Faecalibacterium and Agathobacter was observed in patients with PMS. Additionally, the levels of SCFAs (acetate, propionate, and butyrate) were reduced in the PMS group (42). These findings underscore the high relevance of fiber as a key nutrient in these patients, as children with PMS exhibit a distinct gut microbiota and SCFA profile, which may contribute to the gastrointestinal symptoms and neurodevelopmental issues (developmental delay and autism spectrum disorder-like behaviors) observed in this syndrome (42). Therefore, it is essential to provide nutrition education to children, parents, and caregivers, encouraging the consumption of fibre-rich foods through healthy and appealing recipes.

Moreover, the diet was found to be excessively high in protein (240% of the DRI), indicating a predominance of animal-based sources (meat and processed meat products), a pattern identified through detailed analysis of the 7-day dietary records and food frequency questionnaire. Excessive consumption of animal protein is often associated with high intakes of fat and cholesterol, as well as processed foods. High intake of fats, particularly saturated fatty acids, has been linked to increased oxidative stress, a key factor in mitochondrial dysfunction (43). Notably, individuals with PMS could be more predisposed to mitochondrial dysfunction, a condition that contributes to increased basal oxidative stress. This redox imbalance could be exacerbated by a lipid-rich diet, suggesting a possible synergistic effect between genetic and dietary factors in the worsening of mitochondrial damage in this population (43).

Regarding micronutrients, the intakes of vitamins D and E, folic acid, calcium, and iron were below recommended levels. In particular, vitamin D intake showed a marked deficiency (45% of the recommended amount), suggesting a limited presence in the diet of fatty fish, dairy products, nuts, and leafy green vegetables. Vitamin D deficiency affects bone health by conditioning poor calcium deposition in bone. It would be recommendable to encourage patients’ families to increase their consumption of foods rich in vitamin D (oily fish, egg yolks, dairy products) and try to incorporate them into their daily diet. In addition, encourage sun exposure to synthesize more vitamin D. Deficient calcium intake is coupled with high phosphorus intake, which could be considered potentially detrimental due to its contribution to an imbalance between these two essential nutrients (44, 45). These two minerals play an essential role in phospho-calcium metabolism, neuromuscular function and mineralization (44, 45). This excessive intake of phosphorus could be attributed to a high consumption of meat derivatives or soft drinks, which often contain phosphoric acid as a preservative. This study found that certain patients consumed soft drinks and meat derivatives on a daily basis, not complying with the recommendations. Consequently, PMS patients could benefit from a diet including calcium-rich foods such as dairy products, cruciferous vegetables and nuts, and limiting ultra-processed products rich in phosphorus and its variants. This is relevant considering that the study sample consists of children, and a significant proportion of them are still growing. In addition, a study carried out in a rat model reported that developmental vitamin D-deficiency produces autism-relevant behaviours (46). Given that PMS shares certain neurological and developmental features with autism, vitamin D deficiency may influence brain maturation, immune system function, and gene expression related to social behavior (46–48). Vitamin D deficiency in early life may negatively affect basic cognitive functions such as memory, language, and attention (46–48). In the case of iron, it plays a crucial role in preventing anemia and ensuring the proper synthesis of red blood cells (49). Iron intake should be increased through animal-based sources such as red meat, liver and other offal, fish and seafood, and poultry, as well as plant-based sources including lentils, chickpeas, soy, spinach, pistachios, almonds, pumpkin seeds, and quinoa.

In contrast, intakes of vitamins B1, B3, B12, and C greatly exceeded recommendations (approximately 145–240% of the adequate intake), reflecting a high intake of meats, fortified cereals, and citrus fruits. These elevated vitamin levels generally do not pose toxicity risks, as they are excreted in the urine. Similarly, the minerals magnesium and zinc were elevated, likely due to high meat consumption. No significant differences were found between sexes or age groups in terms of any nutrient, indicating that this dietary pattern is present in both boys and girls with PMS and across all ages. Recent evidence suggests that alterations in zinc status may contribute to both gastrointestinal and neurodevelopmental manifestations of PMS. Although dietary zinc intake in our cohort is not deficient, several studies in humans have shown that a functional zinc deficiency can occur despite apparently adequate intake (50). Patients with PMS exhibit reduced expression of zinc transporters in enterocytes, leading to impaired absorption and systemic zinc deficiency. In addition, zinc is a critical regulator of the SHANK3 protein, whose disruption underlies PMS: synaptic scaffolding by SHANK3 is zinc-dependent, and deficits in zinc availability can exacerbate synaptic dysfunction (51).

The observed nutritional profile (characterized by high intake of sugar and fat-dense foods and low intake of fiber and essential micronutrients) aligns with the comorbidities reported in this disorder: obesity, fatty liver, dental caries, diabetes, behavioral issues, and other metabolic problems (14, 34). Taken together, these results reveal that the diet of children with PMS provides excessive amounts of certain nutrients (sugars, proteins, fats, certain B vitamins) while lacking others that are essential (fiber, vitamins D and E, folic acid, calcium, iron). This dietary imbalance diverges from optimal nutritional patterns and may have a negative impact on their overall health. These findings are consistent with previous studies conducted on healthy Spanish children. It is well established that the average pediatric diet in Spain already tends to exceed the recommended intakes for sugar (52) and fat (53), and that many children show deficiencies in vitamin D and calcium (54). However, in the children with PMS included in this study, these deviations appear to be more pronounced.

In conclusion, nutrition professionals should implement targeted education strategies for children, parents, and caregivers to promote adequate macro- and micronutrient intake. This may include dietary guidance, practical resources on food sources, and, when required, nutritional supplementation in cases of severe deficiencies. Group-based interventions or workshops within the recruiting Phelan-McDermid Syndrome Association could further enhance these efforts.

### 4.4 Limitations of the study and future research

The limited sample size of the study restricts the statistical power and generalizability of the findings; nevertheless, the observations indicate that further research is warranted. In addition, future studies with larger sample sizes should include effect size estimates to complement the analyses.

A significant challenge in this research is the absence of specific DRIs for patients with PMS. These patients exhibit distinct nutritional needs compared to healthy, age-matched children, due to factors such as metabolic alterations, difficulties in nutrient absorption, variations in body composition, and specific requirements for cognitive and neurobehavioural development. The lack of reference values adapted to this population limits the ability to accurately assess nutritional adequacy. This knowledge gap constitutes a limitation of the study and highlights the importance of future work to define specific dietary recommendations for children with PMS. In addition, the lack of a control group prevents determining whether the observed nutritional patterns are specific to PMS or also found in the general pediatric population.

The lack of similar studies conducted in Spain also restricted direct comparison of our findings with existing literature, underscoring the pioneering nature of this investigation into the nutritional intake of Spanish PMS patients. It is crucial to acknowledge that dietary habits can vary significantly within a single country; thus, the diverse geographical origins of our patient cohort should be considered when interpreting results. Consequently, the findings of this study may not be directly generalizable to PMS populations in other countries with different dietary patterns.

Methodological limitations included the dietary-nutritional software used in this study, which did not provide data on simple carbohydrate consumption. Furthermore, it lacked the capacity to determine the average intake of key antioxidant nutrients such as polyphenols, and anti-inflammatory nutrients including omega-3 fatty acids (eicosapentaenoic acid (EPA) and docosahexaenoic acid (DHA)). Given the potential role of these nutrients in mitigating oxidative stress and inflammation, particularly in the context of mitochondrial dysfunction and its influence on neuronal development, their inclusion in future dietary assessments is highly recommended.

Additionally, exposure to sunlight as a source of vitamin D was not assessed, despite the observed low dietary intake of this essential vitamin among the study participants. The lack of biochemical validation of nutrient deficiencies (e.g., serum vitamin D, calcium, ferritin) is a significant limitation of our study. While dietary intake data provide valuable information on nutrient consumption patterns, they cannot conclusively determine the presence of biochemical deficiencies. Future studies should incorporate biochemical markers to more accurately assess nutrient status in this population. It would also be interesting to analyze whether patients use nutritional supplementation in order to determine its impact on nutrient levels.

Consideration should also be given to assessing bone mineral density in these patients, potentially through techniques like Dual-energy X-ray Absorptiometry (DEXA), if deemed appropriate and safe for pediatric populations. This is particularly pertinent given the observed low intake of calcium and vitamin D, unknown sun exposure, and high phosphorus intake.

## 5 Conclusion

Considering the gastrointestinal symptoms observed in Spanish children with PMS, the need for specialized nutritional supervision becomes evident. The involvement of qualified clinical nutrition professionals is essential to develop dietary guidelines tailored to the specific needs of these patients, with the aim of correcting the imbalances observed in this study and thereby improving the quality of life of individuals affected by PMS.

## Data Availability

The original contributions presented in the study are included in the article/supplementary material, further inquiries can be directed to the corresponding author.
